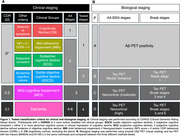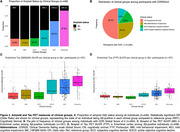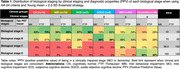# Diagnostic performance of MK and Flortaucipir tau PET for the biological staging of Alzheimer disease

**DOI:** 10.1002/alz70857_107236

**Published:** 2025-12-26

**Authors:** Marina Scop Madeiros, Pamela C.L. Ferreira, Bruna Bellaver, Emma Ruppert, Hussein Zalzale, Carolina Soares, Guilherme Bauer‐Negrini, Guilherme Povala, Andreia Rocha, Matheus Scarpatto Rodrigues, Markley Silva Oliveira, Rayan Mroué, Pampa Saha, Livia Amaral, Firoza Z Lussier, Madeleine Bloomquist, Dana L Tudorascu, Joseph C. Masdeu, David N. Soleimani‐Meigooni, Juan Fortea, Val J Lowe, Hwamee Oh, Belen Pascual, Brian A. Gordon, Pedro Rosa‐Neto, Suzanne L. Baker, Tharick A Pascoal

**Affiliations:** ^1^ University of Pittsburgh, Pittsburgh, PA, USA; ^2^ Houston Methodist Research Institute, Houston, TX, USA; ^3^ Lawrence Berkeley National Laboratory, Berkeley, CA, USA; ^4^ Sant Pau Memory Unit, Department of Neurology, Hospital de la Santa Creu i Sant Pau, Institut d'Investigació Biomèdica Sant Pau (IIB SANT PAU), Facultad de Medicina ‐ Universitat Autònoma de Barcelona, Barcelona, Spain; ^5^ Department of Radiology, Mayo Clinic, Rochester, MN, USA; ^6^ Brown University, Providence, RI, USA; ^7^ Washington University School of Medicine, St. Louis, MO, USA; ^8^ McGill University, Montreal, QC, Canada

## Abstract

**Background:**

The Alzheimer's Association workgroup criteria (AA‐2024) suggest four biological stages based on PET imaging. However, the application of this framework may vary depending on the tau PET tracer used, and the distribution of clinical stages by biological stage may differ. Here, we aim to compare the diagnostic performance of the biological staging of Alzheimer Disease using two different tau PET tracers.

**Method:**

We analyzed 446 individuals and further stratified 167 Aβ‐positive participants from the HEAD study (Longitudinal multicenter head‐to‐head harmonization of tau PET tracers) that underwent both MK‐6240 and Flortaucipir (FTP) tau PET. Four clinical stages (normal, transitional, MCI, and dementia) were defined based on the AA‐2024 criteria. Within stage‐2 we assessed subgroups: subjective cognitive decline (SCD), subtle objective cognitive decline (SOCD), and mild behavioral impairment (MBI). Logistic and linear models corrected by age and sex tested association of groups with amyloid and tau pathology. The biological staging was performed using tracer‐specific thresholds for regional SUVR and testing two regions of interest schemes: (i) proposed by AA‐2024 and (ii) Braak stages (Figure 1). Cohen's weighted‐kappa (K) statistic measured clinical‐biological agreement. Positive predictive value (PPV) of an individual being clinically impaired (MCI or dementia) was calculated for each biological stage.

**Result:**

Clinical stages showed a progressively increased prevalence of Aβ‐positivity and higher entorhinal tau PET pathology from cognitively normal (CN) to SOCD, MCI, and dementia in both tracers. MBI and SCD transitional groups showed no increased Aβ or tau pathology compared to CN (Figure 2). For this reason, only SOCD was considered part of stage 2 for following analyses. All tested biological staging strategies showed moderate agreement (0.34‐0.59) with clinical stages using either tracers. The distribution of biological across clinical stages were similar for FTP and MK, with MK‐6240 showing lower PPV values in earlier stages than FTP (Table 1).

**Conclusion:**

Our results support that AA‐2024 biological and clinical stages show similar clinical‐biological concordance using either Flortaucipir or MK‐6240, although this can vary based on methods tested in the study. The MBI and SCD clinical subgroups showed no difference of pathological burden to clinical stage 1, while SOCD transitional stage showed higher burden compared to stage 1.